# The Impact of Waived Cost-Sharing Policy on COVID-19 Daily Testing and Deaths

**DOI:** 10.7759/cureus.21843

**Published:** 2022-02-02

**Authors:** Ahmed Almuqarrab, Ayman Almuqamam, Fatema Alhayki, Fatimah Alsultan, Mohamed Almuqamam

**Affiliations:** 1 Respiratory Medicine, Pennsylvania State University College of Medicine, Hershey, USA; 2 General Medicine, Salmaniya Medical Complex, Manama, BHR; 3 Family Medicine, Ministry of Health, Manama, BHR; 4 Laboratory Medicine, Penn Medicine Lancaster General Health, Lancaster, USA; 5 Pediatric Critical Care Medicine, Drexel University College of Medicine, Philadelphia, USA

**Keywords:** covid 19 testing, pandemics, deductibles and coinsurance, cost sharing, covid-19

## Abstract

Introduction

Coronavirus disease 2019 (COVID-19) is a public health problem that threatens the world since December of 2019. Several studies demonstrated that enacting waived cost-sharing policies positively impacts the utilization rates of preventative services, but its impact on COVID-19 deaths and tests is largely unknown.

Methods

We hypothesize that applying a waived cost-sharing policy for COVID-19 testing and treatment leads to an increase in the number of COVID-19 tests and a reduction in daily COVID-19 deaths in the State of Michigan. Total test results increase, and total deaths increased were compared pre and post-policy enactment in the State of Michigan and compared to that of the State of Illinois where no such policy existed. Data were obtained from the Coronavirus resources center page at the John Hopkins University of Medicine. A difference in differences approach was employed and linear regression was used to assess data pre and post the policy enactment. Statistical significance was assessed at the 0.05 level.

Results

The state of Michigan had fewer daily COVID-19-related deaths with fewer daily COVID-19 tests than the State of Illinois by 50.19 cases and 28,879 tests respectively. The post-policy period had more daily COVID-19 tests than the pre-policy period by 51,350 tests.

Conclusion

A waived cost-sharing policy for COVID-19 testing and treatment had a positive effect on increasing COVID-19 testing and reducing COVID-19-related deaths at the state level as evident from the experience of two mid-western states.

## Introduction

Coronavirus disease 2019 (COVID-19) is a public health problem that threatens the world since December of 2019 [1]. On March 11, 2020, the World Health Organization announced COVID-19 as a pandemic. The infection strikes all age groups and spreads through large and small droplets from symptomatic and asymptomatic patients [1,2]. According to the Center for Systems Science and Engineering at Johns Hopkins University, there have been more than 115 million cases and more than 2.5 million deaths by March 3, 2021.

In response to this pandemic, different states in the United States have adopted various social and financial policies to keep people healthy and balance the economy. Among various ones, there were two main measures, social distancing actions, and COVID-19 health policy actions. State social distancing covers multiple policies, such as face-covering orders, stay-at-home orders, mandatory travelers quarantine orders, and non-essential business closure followed by phased reopening. The states also enacted various policies under COVID-19 health policy actions including waiving cost-sharing for COVID-19 testing and treatment to ease the financial burden on patients. Further measures included waiving prior authorization requirements, early prescription refill, premium payment grace period, telehealth expansion, along with waiving some of Medicaid, Medicare, and Children's Health Insurance Program requirements. 

Goodwin and Anderson [3] examined the effect of section 4104 of the Affordable Care Act (ACA), which waived cost-sharing of preventive services like mammograms and Pap smears on the utilization rate of such services from 1995 to 2003. The authors found that it had led to an increase in preventative services utilization. Fedewa et al. [4] replicated Goodwin and Anderson’s findings evaluating the utilization rates of colorectal and breast cancer screening especially among patients with low-income, least educated, and Medicare-insured. Carlin et al. [5] studied the effect of cost-sharing elimination on women's contraceptive choices before and after the ACA mandate and found that women are more likely to choose more effective contraceptive methods when cost-sharing drops to zero. More recently, Cook et al. [6] examined the cost-sharing parity on Medicare mental health utilization including psychotropic medication prescriptions and outpatient visits with a similar positive correlation. Conversely, Xu et al. [7] found that reduction of cost-sharing had no significant impact on cancer screening utilization, including colonoscopy, mammogram, and Pap smear.

On September 18, 2020, Michigan State, through the Department of Insurance and Financial Services, announced that the state has an agreement with most of the insurers to waive cost-sharing for coronavirus testing and treatment. It is safe to assume that the financial burden of COVID-19 testing and treatment may hold people back from seeking medical attention and therefore contribute to the rapid spread of infection.

Although many of those studies examined the effect of waived cost-sharing on different preventative measures, no study has examined the effect on COVID-19 prevention. The purpose of this study is to analyze the number of COVID-19 tests and daily COVID-19-related deaths to assess whether the elimination of cost-sharing for COVID-19 testing and treatment was effective in increasing the rate of COVID-19 testing and reducing the daily COVID-19 related deaths among Michigan residents. The authors hypothesize that applying a waived cost-sharing policy for COVID-19 testing and treatment led to an increase in the number of COVID-19 tests and a reduction in daily COVID-19 deaths among Michigan residents.

## Materials and methods

Collected data were obtained from the Coronavirus resources center at the John Hopkins University of Medicine COVID Tracking Project [8]. Data included total test results increase and daily deaths increase for the period of April 2, 2020 to March 7, 2021. Total test results increase was defined as the daily addition of testing numbers of combined positive and negative polymerase chain reaction (PCR) and antigen test results. The daily deaths increase was defined as the daily addition of total fatalities with probable and confirmed COVID-19 diagnosis.

We used the difference in different techniques in the statistical design and ran linear regression to assess data pre and post the policy enactment. Statistical analysis was performed using IBM SPSS version 25.0 (IBM, Armonk, NY, USA) and statistical significance was assessed at the 0.05 level. The examined association was between a quantitative scalar variable (waive cost-sharing policy) and single or multiple descriptive variables (total tests results increase and total deaths increase). Data were collected for two different states: the State of Michigan has enacted the waived cost-sharing policy (treatment state) while the State of Illinois did not (control state). The data are de-identified and publicly available therefore our use of it does not constitute human subjects research that requires IRB review and as defined in 45 CFR 46.102. The state of Michigan enacted the waived cost-sharing policy for COVID-19 testing and treatment on September 18, 2020, therefore, data was collected 170 days before and after that date.

## Results

Table 1 shows the mean increase in deaths and total tests for each state and combined. A total of 680 days represent the number of valid observations for the variables. When combining both groups (Michigan and Illinois), the mean daily death increase was 33.89 (SD=51.469) and the mean total test results increase was 27,352.91 (SD=35,526.84). Each state had 340 days, which represents the number of valid observations for the variables. The mean daily death increase in the State of Michigan was 47.01 (SD=58.15) while the mean total test results increase was 31,114.06 (SD=26,435.12). On the other hand, Illinois's mean daily death increase was 67.27 (SD=55.40), and the mean total test results increase was 54,705.31 (SD=32,051.75).

**Table 1:** Mean increase in deaths and total tests for each state and combined

	Mean	SD
State of Michigan		
Death Increase	47.01	58.156
Total Test Results Increase	31114.06	26435.12
Total number = 340		
State of Illinois		
Death Increase	67.27	55.407
Total Test Results Increase	54705.31	32051.752
Total number = 340		
Combined		
Death Increase	33.89	51.469
Total Test Results Increase	27352.91	35526.846
Total number = 680		

The state of Michigan had fewer daily COVID-19-related deaths than Illinois by 50.19 cases during the study period. This observation could be attributed to the demographic differences among both states. The estimated population, population density, children under five years of age, Black and Hispanic communities are higher in the State of Illinois. Even though the percentage of people over 65 years old in the State of Michigan is higher, the actual number of people over 65 years old in the State of Illinois is higher (2 mils vs 1.7 mils, respectively). Furthermore, there were more daily COVID-19-related deaths in the pre-policy period (April 2 to September 18) than in the post-policy period (September 19 to March 7).

## Discussion

The study analyzed the effect of waived cost-sharing policy on COVID-19 testing and deaths by comparing data from the State of Michigan (treatment group) and the State of Illinois (control group). The state of Michigan was chosen since its government enacted a waiver of cost-sharing of all COVID-19-related testing and treatment on September 18, 2020. Illinois was selected because no such waiver was enacted. The state of Illinois is geographically close to the State of Michigan with relatively similar socio-economic and demographic factors as Table 2 demonstrates [9]. COVID-19-related deaths per state's estimated population are also similar in both states [9].

**Table 2:** Demographics characteristics of States of Michigan and Illinois

Fact	Illinois	Michigan
Population estimates (July 1, 2019), n	12,671,821	9,986,857
Persons under 5 years, %	5.9	5.7
Persons 65 years and over, %	16.1	17.7
Female persons, %	50.9	50.7
White, %	76.8	79.2
Black or African American, %	14.6	14.1
American Indian and Alaska Native, %	0.6	0.7
Asian, %	5.9	3.4
Hispanic or Latino, %	17.5	5.3
With a disability, under age 65 years (2015-2019), %	7.2	10.2
Persons without health insurance, under age 65 years, %	8.6	6.9
Population per square mile (2010), n	231.1	174.8
Land area in square miles (2010), n	55,518.93	56,538.90

Wong et al. [10] found that patients with copays were less likely to seek care compared to patients with no copay even in cases where patients experienced severe symptoms. Waiver of cost-sharing for COVID-19 testing and treatment policy was enacted in the State of Michigan on September 18, 2020. This policy essentially saves patients who need COVID-19 testing or treatment from any out-of-pocket charges, including co-insurance or co-payments. Our data show that after policy implementation, the State of Michigan had a smaller number of daily COVID-19-related deaths than the State of Illinois by 32.95 cases.

Since the beginning of the pandemic, the scientific community firmly believed that COVID-19 testing is an essential step in containing the pandemic. However, not every state was able to offer free and easy access to repeated public testing. Our data indicate that there were more tests during the post-policy period (September 19 to March 7) than during the pre-policy period (April 2 to September 18) 51,350 tests. Despite the waiver, the State of Michigan had fewer daily COVID-19 tests than the State of Illinois by 28,879 during the pre-policy period and 51,349 tests during the post-policy period. That difference could possibly be due to logistical reasons. Larremore et al. [11] noted that the State of Michigan had various logistical issues that affected their testing ability. The state had issues with the supply chain, labs, sample collections, and manufacturing [11].

We recognize that this study has several limitations so the findings should be interpreted with caution. Its retrospective nature introduces inherent biases and limitations. Our data were collected at the state level only. Moreover, many states had concurrently enacted additional nonpharmaceutical interventions, making it difficult to attribute findings to the intervention in question. We did not include data about other state policies such as social distancing, state shutdown, and stay-at-home orders. Lastly, we combined confirmed and probable death in our assessment of daily death increase.

## Conclusions

Waived cost-sharing policy for COVID-19 testing and treatment had a positive effect on increasing COVID-19 testing at the state level as evident from the experience of two mid-western states. Our findings do not support the hypothesis that states with such policies have more COVID-19 testing. Waived cost-sharing policy for COVID-19 testing and treatment correlated positively with reduced daily COVID-19-related deaths. More in-depth assessments of such policies should be carried out at the local and national levels to further understand the observed trends.
